# Wearable EEG Sensor Analysis for Cognitive Profiling in Educational Contexts

**DOI:** 10.3390/s25206446

**Published:** 2025-10-18

**Authors:** Eleni Lekati, Georgios N. Dimitrakopoulos, Konstantinos Lazaros, Panagiota Giannopoulou, Aristidis G. Vrahatis, Marios G. Krokidis, Panagiotis Vlamos, Spyridon Doukakis

**Affiliations:** Bioinformatics and Human Electrophysiology Laboratory, Department of Informatics, Ionian University, 49100 Corfu, Greece; dimitrakopoulos@ionio.gr (G.N.D.); vlamos@ionio.gr (P.V.); sdoukakis@ionio.gr (S.D.)

**Keywords:** wearable EEG, signal analysis, cognitive profiling, neurophysiological monitoring, personalized education

## Abstract

Electroencephalography (EEG) provides a powerful means of capturing real-time neural activity, enabling the study of cognitive processes during complex learning tasks. This study explores the application of wearable EEG and advanced signal analysis to examine cognitive profiles of 30 sixth-grade students engaged in fraction learning. Using validated estimations alongside interactive digital tools such as Fraction Lab and the Diamond Paper task, EEG recordings were processed to evaluate spectral dynamics across delta, theta, alpha, and beta bands. Results revealed that lower-performing students exhibited elevated delta and theta power under cognitive load, whereas higher-performing students showed more stable beta activity linked to cognitive control. These findings highlight the utility of EEG-based signal analysis for identifying neurocognitive markers associated with conceptual and procedural knowledge (PK) in mathematics. The integration of such methodologies supports the development of precision-oriented educational strategies grounded in objective neural data. Clustering further revealed three learner profiles: Core Support Needed, Developing, and Advanced, while classification analyses confirmed that EEG features, especially gamma and beta oscillations, reliably distinguished among them, underscoring the potential of neurocognitive markers to guide adaptive instruction.

## 1. Introduction

Fractions are foundational for later mathematics and everyday problem solving, and persistent difficulties can hinder progression to advanced topics and affect academic and career outcomes [1,2,3,4,5]. Challenges with fractions relate to later performance in algebra and geometry [6,7]. Many students struggle with equivalence, number-line placement, and operations—partly because fractions behave differently from whole numbers [3,8,9,10,11,12,13]. These difficulties are also shaped by cultural context, teaching practices, teachers’ knowledge and beliefs, and curricular differences across systems [14,15,16,17].

Recent work argues for personalized instruction that adapts to individual learner needs [18,19,20,21] and for adaptive learning environments that better prepare students for rapidly changing demands [22,23,24]. To inform such environments, several methods monitor learners’ cognitive states during mathematics. Classroom observation and performance-based assessments provide indirect insights with limited neural specificity, while eye-tracking reveals visual attention and strategy use in fraction tasks but does not directly index mental effort or neural engagement [25,26]. fMRI has identified key regions for symbolic and non-symbolic number processing (e.g., IPS, prefrontal cortex) but is impractical for real-time or classroom use due to low temporal resolution and logistical constraints [27,28,29].

By contrast, wearable/wireless EEG affords real-time, continuous, non-invasive monitoring. ERP-based EEG studies have distinguished neural dynamics between fractions and decimals and mapped the temporal course of cognitive engagement in numerical tasks [30], positioning EEG as a candidate for integrating cognitive monitoring with personalized instruction in authentic school settings. Building on this, wireless EEG systems can capture brain activity in real time and flag moments of confusion or disengagement so that teachers or adaptive systems can respond promptly [31].

When integrated with intelligent platforms, such data can guide adjustments to strategy, pacing, or content—particularly valuable in fractions, where misconceptions often persist—supporting earlier detection of bottlenecks and more tailored assistance for improved conceptual understanding and retention [32]. In practice, wearable educational technologies enable non-invasive, continuous collection of neural/physiological data in real classrooms to estimate engagement, mental workload, and frustration [33].

Beyond education, wearable sensing spans biomedical, industrial, and human-performance applications and includes bioinspired force sensors and deep-learning pipelines for context-aware feedback [34,35,36,37,38]. In this study, however, we focus specifically on wearable EEG during classroom fraction learning.

In recent years, a growing number of wearable electroencephalography (EEG) systems have been developed, offering compact, user-friendly, and mobile solutions for monitoring brain activity outside traditional laboratory settings. Devices such as Muse 2 [39], Dreem [40], Cognionics [41], and Kernel Flow [42] represent prominent examples of this trend, each combining portability with advanced signal acquisition capabilities. These systems enable continuous and unobtrusive data collection, supporting applications in cognitive neuroscience, clinical monitoring, neurofeedback, and brain–computer interface research. By lowering barriers to accessibility and facilitating real-world neuroimaging, wearable EEG technologies are reshaping the landscape of brain research and expanding opportunities for both scientific and translational applications.

This work extends prior classroom EEG research by focusing on sixth-grade fraction learning in situ, explicitly contrasting conceptual and procedural task demands, and triangulating band-limited spectral features with multiple validated assessments (RODI, Fraction Lab, Diamond Paper). Unlike earlier studies emphasizing aggregate “attention/load,” we provide signal-level spectral analysis aligned to curriculum-relevant competencies in younger learners.

### 1.1. Conceptual Understanding (CU) and Procedural Knowledge (PK) of Fractions

The study of fractional understanding in mathematics education emphasizes the importance of both conceptual understanding (CU) and procedural knowledge (PK), recognizing these components as equally vital for achieving mathematical proficiency [43,44]. CU refers to the comprehension of core mathematical concepts and principles within a domain [45,46], as well as the ability to grasp the rationale behind mathematical procedures [47,48,49]. Within the domain of fractions, CU involves an understanding of the unique relationship between the numerator and the denominator and the implications this relationship holds for mathematical reasoning [48,50].

PK, on the other hand, pertains to the ability to perform mathematical procedures and to understand the steps and operations required for their execution [51,52]. In the context of fractions, PK is notably complex and distinct from operations involving whole numbers. Despite their differences, both CU and PK are widely regarded as essential to the learning and mastery of fractions [43,44]. Nonetheless, previous research has frequently prioritized one form of knowledge over the other [53,54,55,56], often advocating for the primacy or earlier development of either CU or PK [51,52]. Still, scholars concur that a clear distinction between CU and PK, both in theory and in instructional practice, is necessary for advancing the teaching and learning of fractions [14,53,55,57,58].

Over the past decade, investigations into CU and PK of fractions have predominantly employed group-level analyses, which tend to obscure the individual pathways through which these types of knowledge develop, as well as the multitude of factors that influence their formation [21]. In response to this limitation, recent research has shifted toward examining CU and PK at the individual level, seeking to uncover the diversity in students’ learning trajectories [48,59]. These studies have shed light on the personalized strategies students adopt in acquiring CU and PK of fractions, revealing considerable variability in the developmental processes involved. Such findings support the view that CU and PK may evolve independently, thereby challenging traditional assumptions about a linear progression of mathematical learning [21].

A recent review further enhances the research community’s growing acknowledgment of the distinct developmental paths of CU and PK in the context of fractions [19]. The review also highlights a contemporary shift in the field towards more nuanced investigations, which examine CU and PK within specific cognitive domains and from an individual-centered perspective. By recognizing the differentiated influence of students’ cognitive characteristics and the nature of the fractional concepts studied, this body of work underscores the intricacies involved in disentangling CU from PK [19].

### 1.2. Neurocognitive Basis of Fraction Processing

In recent years, increasing attention has been directed toward identifying the cognitive mechanisms that underpin the processing and learning of fractions. Although individuals can mentally represent fraction magnitudes, ratios, and proportions, symbolic fractions introduce unique difficulties that often lead to systematic biases and errors across both student and adult populations. However, the precise stages at which these difficulties emerge remain inadequately understood [29].

Advances in contemporary psychophysiology have established a strong link between individual patterns of brain electrical activity, as measured by electroencephalography (EEG), and cognitive load as well as performance outcomes [60,61]. While prior neuroimaging research has demonstrated that the processing of fractions and decimals involves overlapping yet distinct brain regions [62], the temporal dynamics of how these numerical formats are processed remain underexplored.

To address this, Lin et al. [63] used event-related potentials to manipulate notation and numerical distance and found that decimals elicited larger N1/smaller P1 responses in parietal areas compared to fractions. In contrast, numerical distance significantly influenced the fronto-central P2 for fractions, whereas a left-anterior N2 carried this effect for decimals, indicating distinct early processing pathways across formats.

Further insights into the neurocognitive mechanisms of fraction processing were provided by Rivera and Soylu [30], who also used EEG to explore how adults comprehend the magnitudes of fractions. Their results showed longer reaction times for mismatches and ERP signatures (frontal N270 to mismatches, parietal P300 to matches), suggesting overlap with target-detection/conflict mechanisms known from arithmetic.

Despite these advances, a notable gap persists concerning the neural correlates of fraction processing in children—a population for whom the development of fraction understanding is particularly critical. While much of the current research focuses on adults, children in primary education represent a key demographic for examining the emergence of fraction concepts.

Park et al. [28] showed a progression from sensitivity to non-symbolic ratios toward understanding symbolic fractions, shaped by educational experiences. Applying EEG to this developmental shift can reveal when and how fraction representations consolidate in childhood, enabling better alignment of instructional strategies with cognitive development and informing more effective, developmentally appropriate interventions.

### 1.3. Theoretical Framework and Research Questions

Building upon the aforementioned findings, the present study sought to integrate a range of research tools alongside neurophysiological measurements in order to investigate individualized student profiles. These profiles were characterized according to students’ cognitive abilities, their levels of conceptual understanding (CU) and PK in fractions, and their corresponding neurophysiological patterns.

In the Greek educational context, the instruction of fractions is predominantly completed by the end of the sixth grade in primary school. While a limited set of additional topics are introduced in the first year of middle school, the focus at that stage is placed almost exclusively on procedural aspects. Prior research in Greece has indicated that notable individual differences in fraction comprehension, first observed among first-year middle school students, persist through the third year of middle school [24]. These findings underscore the importance of examining CU and PK in students at the conclusion of primary education and raise questions as to whether these differences stem from students’ varying levels of experience, distinct cognitive profiles [48], or characteristics specific to the Greek educational system and broader contextual influences.

To advance this line of inquiry, this study integrates a series of tools designed to evaluate students’ cognitive and mathematical learning profiles. These include the RODI test for assessing cognitive abilities, as well as tasks that measure conceptual understanding and PK of fractions. Additionally, the study employs Fraction Lab and the Diamond Paper—two complementary tools aimed at gauging students’ applied understanding of fractions. By combining these assessments with real-time EEG recordings, the research seeks to capture a holistic view of each learner’s cognitive and neurophysiological engagement.

In line with these aims, the research objectives are articulated as follows:

- To examine sixth-grade students’ levels of conceptual understanding and PK of fractions, as well as their performance on applied tasks such as Fraction Lab and the Diamond Paper, in order to build a comprehensive picture of each learner’s mathematical profile.
- To classify students into distinct cognitive and neurophysiological profiles using data from the RODI test, EEG recordings, and performance assessments, thereby enabling the identification of learning patterns and needs.
- To explore how these individualized profiles can inform the development of personalized learning pathways, with the goal of tailoring educational interventions to optimize engagement, comprehension, and long-term retention in mathematics.

## 2. Materials and Methods

### 2.1. Participants

EEG data were collected from a sample of 30 sixth-grade students (15 females), with a mean age of 11.6 years, drawn from six different primary schools within the Municipality of Argos-Mykines (2nd, 3rd, 4th, and 6th Argos Primary Schools, Kefalari Primary School, and Maltezos Private Schools). All participating schools adhered to the same national curriculum and maintained a relatively comparable student performance level. The selection of students was conducted randomly, and none of the participants had been diagnosed with learning difficulties.

The study took place in a private setting, with participation contingent upon the provision of signed parental consent forms. During testing sessions, students completed each task individually and the supervisor monitored the process remotely through a computer connected through TeamViewer. Each session lasted between 45–68 min, ensuring sufficient time for the completion of all activities and observations. All students answered the same questions in all tests; the variation in completion time depended on the time each student needed to respond.

All data were collected in individual, one-on-one sessions. For pedagogical reasons, the Diamond Paper was administered after Fraction Lab within the same appointment; counterbalancing of task order was therefore not feasible in this cohort. Scheduling constraints in the school context also limited the possibility of arranging a second counterbalanced appointment.

### 2.2. Research Tools

For the purposes of this study, three research tools were employed, each previously validated for reliability and effectiveness. Additionally, a newly proposed tool was utilized to assess students’ CU and PK in an integrated manner.

1. **Assessment of Students’ Cognitive Abilities**: Students’ cognitive abilities were evaluated using the “RODI TEST”, a tool approved by the Ethics and Deontology Committee of the Ionian University under protocol number 3600/13-09-2022. Demographic information was also collected as part of this assessment.
2. **Evaluation of Conceptual (CU) and Procedural Knowledge of Fractions (PK)**: The evaluation of students’ CU and PK regarding fractions involved the use of multiple research instruments:
  - A validated 25-item tool was used to assess sixth-grade students’ procedural and conceptual understanding of fractions [24]. It includes ten items targeting procedural skills and fifteen multiple-choice items designed to evaluate conceptual understanding while minimizing reliance on procedures. The tool demonstrates strong psychometric properties (CVI = 1; Cronbach’s α = 0.921 for procedural, 0.731 for conceptual tasks) and was administered via Google Forms. Results were analyzed separately for each section, offering a reliable measure of students’ fractional competence  (Link to Test).
  - Fraction Lab, developed by Mavrikis et al. at University College London (UCL), is a research-validated digital platform designed to enhance both conceptual and procedural understanding of fractions [64,65]. It combines exploratory activities and structured exercises, allowing students to visualize fractions through number lines, area models, and dynamic partitioning. In this study, Fraction Lab assessed fraction equivalence and ordering, with all interactions securely recorded  (Link to Test).
  - Diamond Paper, a versatile tool inspired by Boaler’s framework [66,67], and developed following recommendations by Cathy Williams, enables students to interpret fractional relationships using four distinct approaches. Adapted from the widely recognized Diamond Template, it prompts students to create a narrative, draw a sketch, represent fractions with objects (all targeting conceptual understanding), and perform a calculation (PK). Each student completed an A4 sheet folded into a diamond shape, centered on a given fraction relationship, with responses later scanned, graded, and securely stored.

The study was conducted in a private setting, with participation contingent upon the provision of signed parental or guardian consent. Direct researcher intervention was limited to the third part of the session, where an explanation on how to use the digital platform was necessary. The total duration of each session ranged from 45 to 68 min.

### 2.3. Collection and Pre-Processing of EEG Data

**Figure 1:**
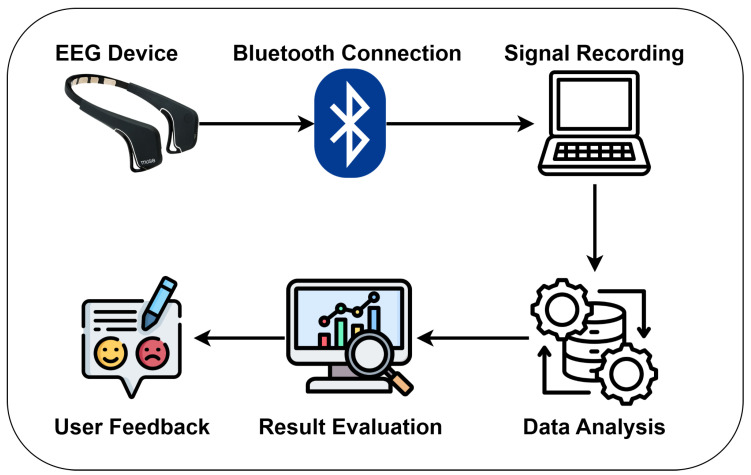
Schematic representation of the EEG data acquisition and analysis pipeline using the Muse 2 device. Electrophysiological signals are recorded through the Muse 2 headband and transmitted via Bluetooth Low Energy (BLE) to an edge node (laptop or tablet). The data are then processed using dedicated analysis modules (e.g., EEGLAB plugin or MNE). Results are evaluated and interpreted, with insights fed back to the user, enabling an iterative loop of neurofeedback and performance assessment.

During the administration of the tests, neurophysiological data were recorded using the Muse 2 headset (Figure 1), a compact, Bluetooth-enabled device designed for portable brain activity monitoring. This wearable system captures a range of electrophysiological signals, including electroencephalography (EEG), heart rate, and head movement via an integrated accelerometer. Data were securely transmitted in real time to a local device, such as a computer or tablet, where they were encrypted and stored to ensure participant confidentiality.

The Muse 2 employs four dry EEG sensors positioned at AF7 and AF8 over the frontal cortex, and TP9 and TP10 over the temporal lobes. These locations allow for effective monitoring of brain regions associated with attention, working memory, and numerical cognition. A reference electrode located at FPz supports stable signal acquisition. The system samples at a frequency of 256 Hz, enabling the detection of rapid changes in neural activity, including early event-related potential (ERP) components.

Wireless communication between the Muse 2 and the recording device is achieved through Bluetooth Low Energy (BLE), which ensures low-latency and energy-efficient data transmission. This wireless design reduces motion-related artifacts and removes the constraints of traditional wired EEG systems, creating a more natural and comfortable experience for participants. The overall setup facilitates seamless EEG signal acquisition in mobile and experimental settings, making it well-suited for educational and applied research without the logistical burdens of conventional laboratory equipment.

EEG data processing was carried out using the EEGLAB toolbox in MATLAB (2022b) (MathWorks Inc., Natick, MA, USA). Standard pre-processing steps were applied [68]. The data were first band-pass filtered between 1 and 45 Hz and then re-referenced to the average. Artifacts were automatically removed using the Artifact Subspace Reconstruction (ASR) method with default parameters. Subsequently, the power spectral density was calculated across standard frequency bands: delta (1–4 Hz), theta (4–7 Hz), alpha (8–12 Hz), beta (13–20 Hz), and gamma (20–45 Hz). Visualization of the processed results was performed using the MNE-Python package [69]. Figure 1 provides a graphical abstract of the methodlogy used for this analysis

## 3. Results

**Figure 2:**
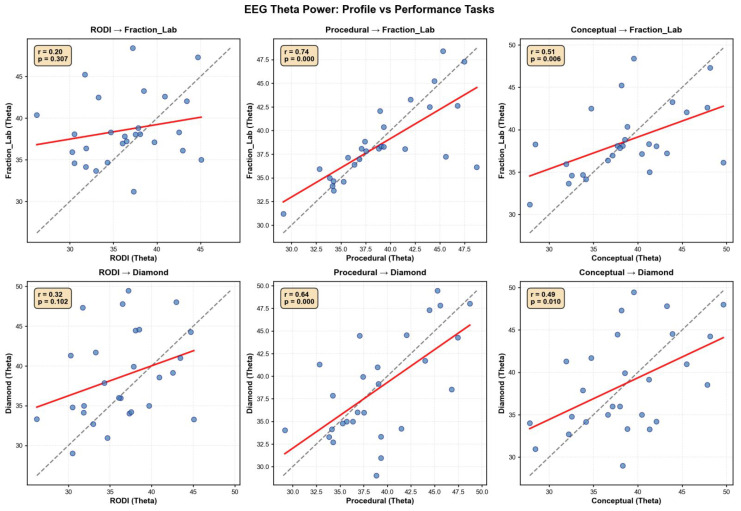
Correlations between cognitive profile and task-related EEG theta power. Scatter plots display the relationship between mean EEG theta power (4–7 Hz) during profile tasks (RODI, PK, CU) and performance tasks (Fraction Lab, Diamond). Each dot represents a participant. The red line shows the linear regression fit, and the dashed gray line represents the identity line (y = x). Positive correlations indicate that individuals with greater theta activation during cognitive profiling also exhibit higher engagement during task execution.

To assess whether students’ cognitive profiles were reflected in their neurophysiological engagement during task execution, we analyzed the relationship between EEG theta power during profile tasks and fraction-based activities. As shown in Figure 2, significant positive correlations were observed in several comparisons, particularly between Conceptual Knowledge and Fraction Lab, suggesting that learners with stronger conceptual engagement also exhibited increased theta power when solving applied tasks. The presence of a regression line above the identity line in most plots indicates that, for many students, theta activation was higher during performance than during profiling.

**Figure 3:**
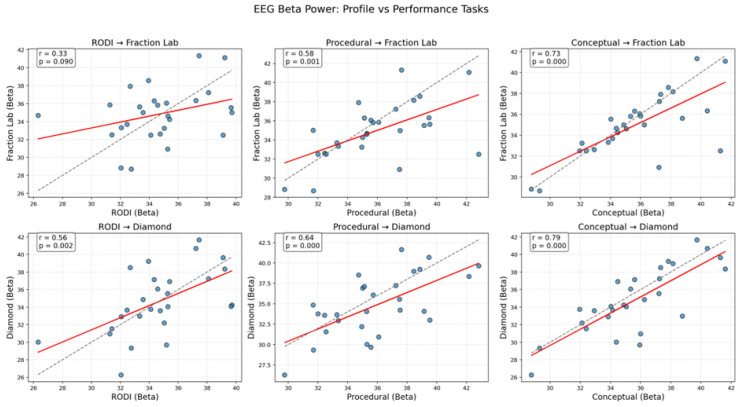
Associations between cognitive traits and EEG beta power during performance. Similar to Figure 2, these scatter plots illustrate the relationship between EEG beta power (13–30 Hz) in cognitive profile conditions and fraction-based task performance. Stronger beta activation in profile tasks is associated with enhanced beta responses during task execution, suggesting consistent cognitive processing dynamics across contexts.

A similar pattern emerged when examining beta power (Figure 3), which is typically linked to cognitive control and mathematical reasoning. Students who demonstrated higher beta activity during conceptual or procedural profiling also tended to exhibit elevated beta responses during performance in the Fraction Lab and Diamond tasks. This pattern was most pronounced for the Procedural–Diamond pairing, supporting the notion that PK may directly facilitate performance under complex task demands.

The EEG data were analyzed using ANOVA with the aid of the open-source software PSPP 2.0  (found here) to identify statistically significant differences across conditions. Only results that reached statistical significance are presented and discussed. Specifically, among all the values examined, three cases yielded statistically significant differences, with *p*-values less than or equal to 0.05 (see Table 1). For these cases, a post hoc t-test with Bonferroni correction was conducted to determine which conditions differed significantly, as reported in the final column of Table 1.

Based on the results presented in Table 1, distinct patterns of brain activity were observed in the AF7 electrode site across different frequency bands and test conditions.

In the delta band, statistically significant differences emerged when comparing test a with both tests b_2 and d. Specifically, test b_2 exhibited a significantly higher mean power value than test a, indicating enhanced neural activation during this condition. Similarly, test d also showed a significantly greater mean value compared to test a. These findings suggest that both test b_2 and test d elicited stronger low-frequency brain responses, potentially reflecting increased cognitive effort or engagement during these tasks.

In the theta band, a comparable trend was observed. Test d produced significantly higher mean values in the AF7 channel relative to test a, further supporting the notion of elevated cognitive processing demands or attentional load associated with this specific task.

It should also be noted that, in the alpha band, the RODI test yielded noticeably lower mean power values in AF7 compared to tests b_2 and d. This decrease in alpha activity may reflect reduced inhibition or heightened cortical activation during the RODI task relative to the others. Collectively, these spectral differences underscore the sensitivity of the AF7 site in capturing task-related neural dynamics and provide evidence of differential engagement across the administered test conditions.

**Figure 4:**
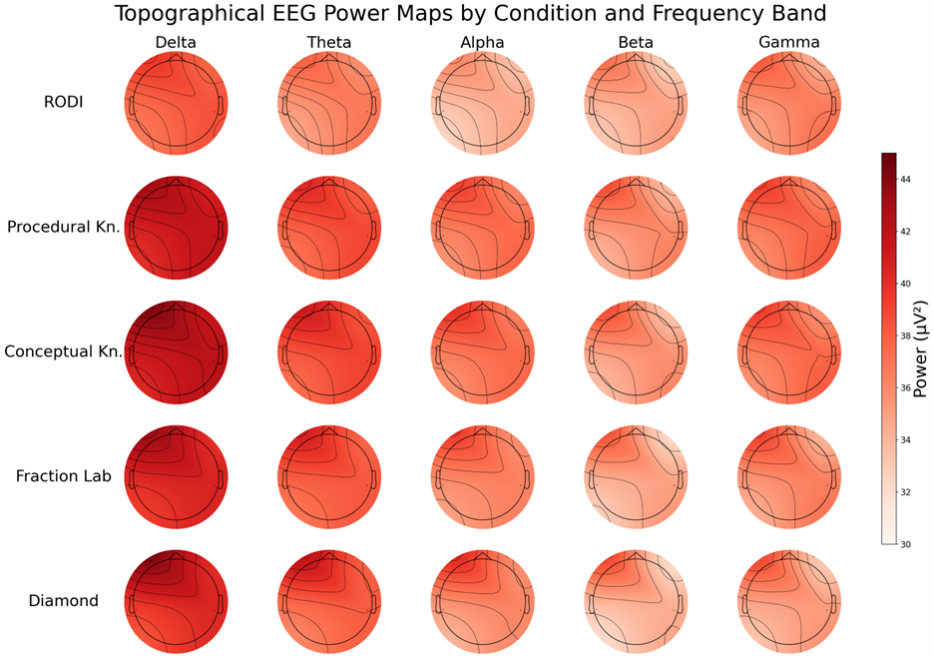
Topographical EEG power maps across five cognitive task conditions (RODI, Procedural Knowledge, Conceptual Knowledge, Fraction Lab, and Diamond) and five frequency bands (Delta, Theta, Alpha, Beta, Gamma). A consistent pattern is observed in the delta and theta bands, where lower-performing students exhibit elevated power, particularly during Procedural and Conceptual Knowledge tasks. This heightened low-frequency activity may reflect increased cognitive effort or difficulties in sustaining attention. In contrast, higher-performing students show more moderate delta and theta power, indicative of more efficient neural processing and attentional control during task execution.

The panoramic EEG visualization found in Figure 4 provides a detailed representation of how different students engage with a series of mathematical tasks at the neurophysiological level. Each row corresponds to a distinct learner profile, while each column represents a specific frequency band (delta, theta, alpha, beta, gamma) measured during five tasks: RODI, PK, CU, Fraction Lab, and Diamond. This layout enables direct comparisons of cortical activation across cognitive conditions and student profiles.

A pattern emerges in the delta and theta bands, where students with lower academic and cognitive performance display elevated power across most tasks, particularly during Procedural and Conceptual Knowledge activities. These increases in low-frequency activity are often associated with greater cognitive effort or difficulty maintaining attention, suggesting that they may be using more neural resources to complete the tasks. In contrast, higher-performing students exhibit more moderate delta and theta power, which may indicate more efficient and focused neural processing during task performance.

It is also important to note that the Diamond test was conducted last in the sequence of tasks, which may have contributed to observable changes in brain activity—particularly among lower-performing students. Increased delta and theta power in this final test, coupled with reductions in higher-frequency bands, may reflect signs of mental fatigue, reduced focus, or cognitive disengagement due to the cumulative demand of previous tasks. This temporal factor likely exacerbated performance disparities, especially for students with lower cognitive stamina or attentional control.

These patterns highlight how learner profiles are linked to distinct neural activation signatures during mathematical processing. High-performing students tend to show balanced activity across frequency bands, reflecting focused engagement and cognitive efficiency, whereas students with lower performance rely more heavily on lower-frequency oscillations, often associated with increased effort or cognitive strain. The findings also point to the cognitive demands of the task types themselves, with fraction-related activities—especially those involving conceptual understanding—eliciting greater neural activation.

**Figure 5:**
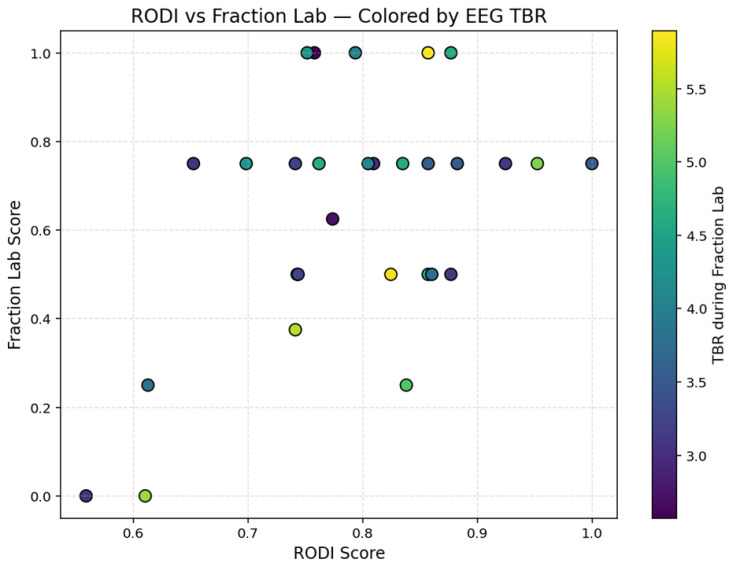
Scatter plot illustrating the relationship between RODI scores and performance in the Fraction Lab task, with each data point colored according to the subject’s Theta/Beta Ratio (TBR) measured during the task. Lower TBR values (blue) indicate increased cognitive focus, whereas higher TBR values (yellow) may reflect increased cognitive load or reduced attentional control.

A scatter plot is also used to explore the relationship between students’ cognitive profile (as measured by the RODI score) and their performance in the Fraction Lab task. Each data point was color-coded based on the participant’s Theta/Beta Ratio (TBR) during the task. As shown in Figure 5, students with higher RODI scores generally achieved higher scores in the Fraction Lab, indicating a positive association between cognitive potential and task success. Notably, several high-performing students also exhibited relatively low TBR values, suggesting a more focused neurocognitive state during task execution. The Theta/Beta Ratio (TBR) was included as a neurophysiological index of attentional control during the Fraction Lab task, with lower TBR values typically associated with higher cognitive focus and reduced distractibility [70,71].

The identification of distinct learner profiles represents a critical step toward personalized education. In this analysis, we employed unsupervised machine learning techniques to classify sixth-grade students into meaningful cognitive and performance-based categories using three well-established assessment instruments: the RODI test for cognitive abilities, a validated measure of Conceptual Understanding (CU) of fractions, and a measure of Procedural Knowledge (PK) in fraction operations. These three metrics have been extensively validated in educational research and collectively provide a comprehensive representation of a student’s mathematical learning profile, capturing both their underlying cognitive capacity and their domain-specific knowledge in two complementary dimensions.

**Figure 6:**
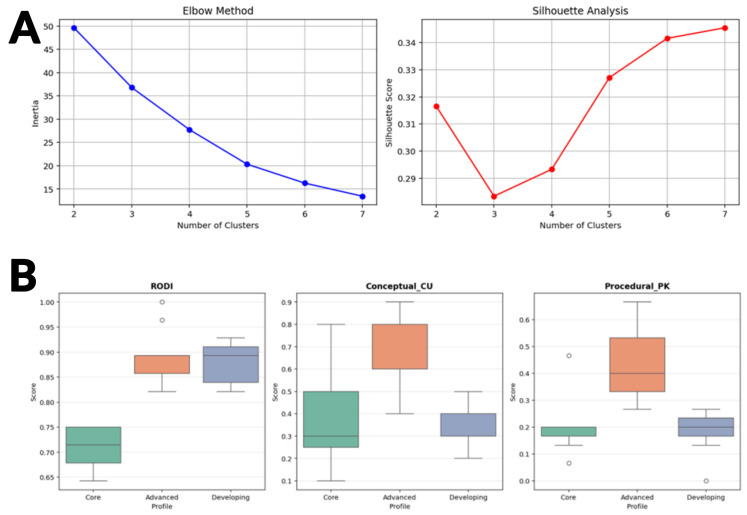
Cluster validation and student profile characterization. (**A**) Elbow and Silhouette analyses both indicate k = 3 as the optimal solution, balancing compactness, separation, and interpretability. (**B**) Boxplots of assessment scores confirm hierarchical differentiation, with RODI showing the strongest separation and content knowledge revealing greater within-group variability and notable outliers. Heatmap of cluster centers summarizes profile characteristics: Advanced Learners score highest across all measures, Core Support Needed show relative cognitive strength but weak content mastery, and Developing Learners display strong cognitive ability with lower procedural knowledge.

**Figure 7:**
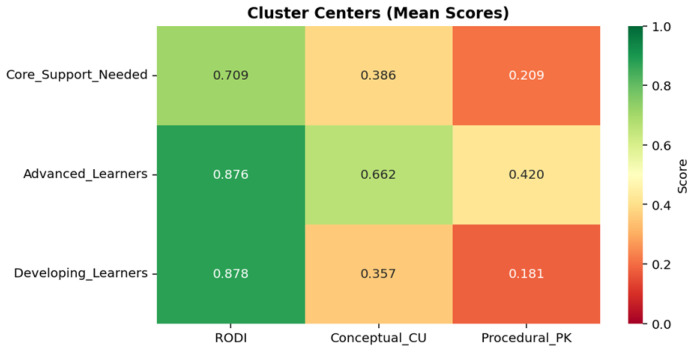
Heatmap of cluster centers summarizes profile characteristics: Advanced Learners score highest across all measures, Core Support Needed show relative cognitive strength but weak content mastery, and Developing Learners display strong cognitive ability with lower procedural knowledge.

K-means clustering was applied to identify natural groupings within the student population based on their performance across these three dimensions. The decision to extract three clusters was not arbitrary but rather was informed by both statistical validation metrics and pedagogical expertise. From a technical standpoint, both the Elbow Method and Silhouette Analysis supported the three-cluster solution as optimal for this dataset (Figure 6 and Figure 7).

**Figure 8:**
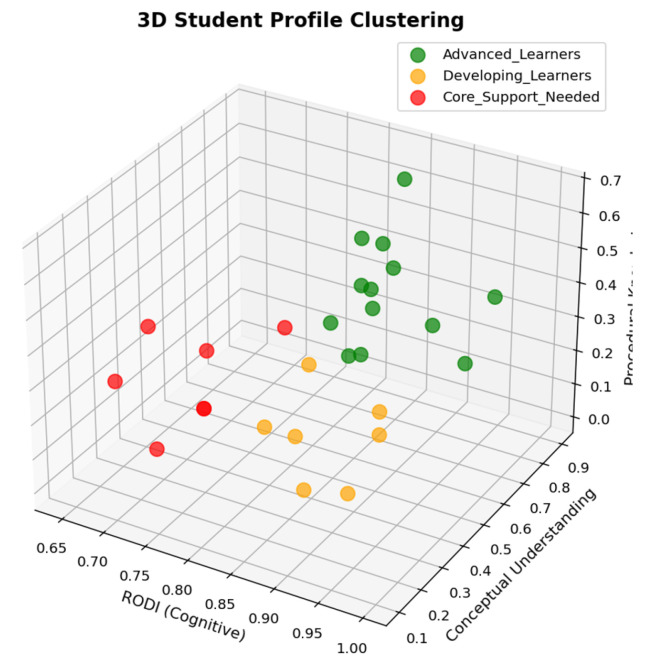
Three-dimensional scatter plot of RODI cognitive ability, Conceptual Understanding, and Procedural Knowledge shows clear spatial separation: Advanced Learners (green) with high scores, Core Support Needed (red) with low performance, and Developing Learners (orange) in between.

More importantly, from an educational neuroscience perspective, this three-tier classification aligns with established frameworks in differentiated instruction and reflects the heterogeneity commonly observed in classroom settings. Educational experts and specialists in brain activity patterns would independently arrive at similar categorical distinctions when characterizing learners: those who demonstrate advanced mastery across cognitive and content domains, those who are developing and show moderate performance, and those who require additional support due to foundational gaps. The convergence of data-driven clustering results with expert pedagogical judgment provides strong validation for this classification approach and suggests that these profiles represent meaningful and actionable student categories (Figure 8).

Having established three distinct student profiles through unsupervised clustering, the subsequent phase of this research focuses on developing predictive models capable of automatically classifying students into these profiles based solely on their neurophysiological signatures during task performance. This classification approach addresses a critical practical question: can real-time brain activity patterns captured during mathematical problem-solving tasks serve as objective biomarkers for identifying a student’s cognitive and learning profile? Rather than relying exclusively on traditional assessment scores, which provide post hoc snapshots of performance, EEG-based classification offers the potential for continuous, non-invasive monitoring of learner states during actual task engagement. This methodology could ultimately enable adaptive educational systems to respond dynamically to students’ neurophysiological indicators, adjusting instructional content, pacing, and support in real time based on detected cognitive patterns.

The classification models were trained using EEG data collected from two interactive digital learning environments (conditions c and d), specifically the Fraction Lab and Diamond Paper tasks. These conditions were selected as feature sources because they represent authentic mathematical problem-solving contexts where students actively engage with fraction concepts, in contrast to the passive assessment conditions used for profile determination (RODI, Conceptual Understanding, and Procedural Knowledge tests). Brain activity was recorded across four scalp locations (TP9, AF7, AF8, TP10) spanning temporal and frontal regions, with power spectral density computed for five canonical frequency bands: delta (1–4 Hz), theta (4–7 Hz), alpha (8–12 Hz), beta (13–20 Hz), and gamma (20–45 Hz).

**Figure 9:**
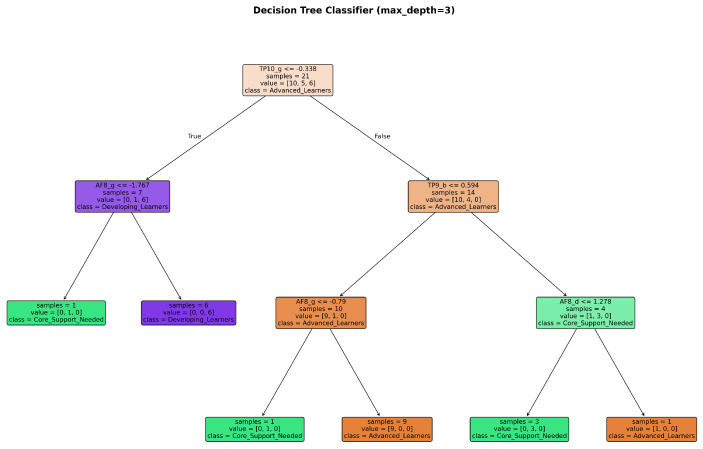
Decision Tree visualization shows that three branching rules—primarily involving right temporal gamma (TP10_g), right frontal gamma (AF8_g), and left temporal beta (TP9_b)—suffice to distinguish the three profiles, highlighting gamma oscillations as dominant discriminators.

**Figure 10:**
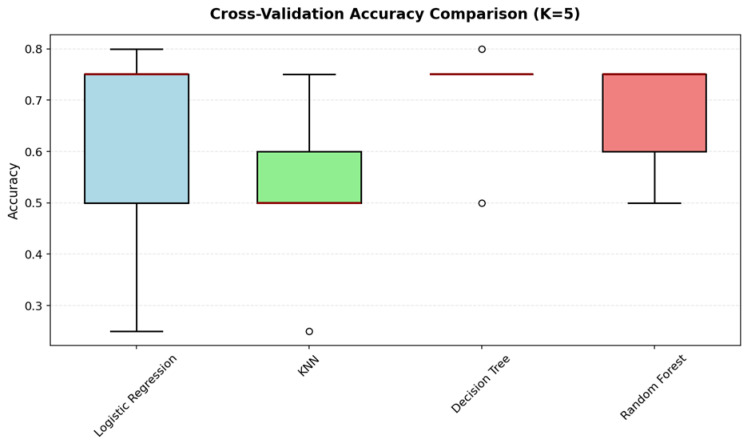
Cross-validation accuracy across four algorithms (Random Forest, Logistic Regression, KNN, Decision Tree) reveals Random Forest as the most consistent and accurate method, while Logistic Regression shows greater variability due to the non-linear nature of EEG features.

**Figure 11:**
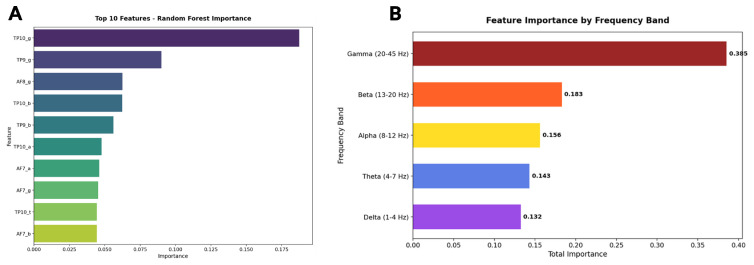
(**A**) Random Forest feature importance ranking identifies TP10_g as the most informative EEG marker, followed by other temporal and frontal features, with gamma and beta power emerging as key predictors of profile differentiation. (**B**) Aggregated feature importance by frequency bandconfirms gamma activity as the strongest contributor, followed by beta and alpha, with theta and delta playing smaller but non-negligible roles. Together, these results show that neurophysiological differences underlying student profiles are most strongly reflected in high-frequency cortical oscillations linked to attention, integration, and executive control.

This yielded 20 neurophysiological features per student, capturing the multidimensional signature of cognitive engagement across different neural oscillatory rhythms. Four supervised learning algorithms were trained and evaluated using stratified five-fold cross-validation to ensure robust performance estimation despite the modest sample size: Logistic Regression (as an interpretable linear baseline), K-Nearest Neighbors (a non-parametric instance-based approach), Decision Tree (for transparent rule extraction), and Random Forest (an ensemble method balancing accuracy with interpretability through feature importance analysis). The classification framework thus bridges neuroscience and machine learning to establish whether patterns of cortical activity during fraction learning tasks contain sufficient information to reliably infer students’ broader cognitive and performance profiles (Figure 9, Figure 10 and Figure 11).

## 4. Discussion

This study highlights the potential of neurophysiological data to uncover meaningful differences in how students engage with mathematical tasks involving fractions. The EEG findings align with previous research showing that fraction processing draws on brain regions associated with attention, working memory, and numerical reasoning. Differences in power spectral density across frequency bands and task types reflect varying levels of cognitive effort and engagement among students.

Additionally, the observed relationship between RODI and Fraction Lab performance supports the view that students with stronger cognitive profiles—especially in areas such as metacognition and working memory—are better prepared to engage with mathematics learning tasks. The use of EEG-derived TBR values adds another layer of insight: lower TBR values, indicative of enhanced attentional control, were frequently associated with stronger task performance. This observation echoes prior research linking reduced TBR with heightened cognitive engagement. Altogether, these findings underscore the value of integrating behavioral and neurophysiological data to better understand individual learning differences. Associations with theta activity suggest a continuity between cognitive potential and task-based engagement. Theta power has long been linked to working memory and cognitive control. The observation that students with elevated theta activation during cognitive profiling also exhibited higher theta during task execution reinforces the idea that cognitive readiness translates into real-time engagement during learning. This neurophysiological pattern provides a tangible link between latent cognitive traits and active performance.

Beta power, commonly associated with analytical problem-solving, planning, and sustained attention, showed consistent activation across both profiling and task phases. This consistency suggests that certain learners may exhibit a stable mode of cognitive control that extends from inherent reasoning ability to its practical application. Such neurophysiological stability may serve as a marker of cognitive resilience or learning efficiency, aligning with prior research that connects beta oscillations to mathematical achievement. The observed correlations offer key insights into how different cognitive profile components relate to students’ performance in digital learning environments. The strongest link was found between RODI and Fraction Lab outcomes, reinforcing the idea that general cognitive skills—particularly attention regulation and metacognitive ability—support better engagement and learning in mathematics. A moderate correlation between conceptual knowledge and both outcomes suggests that understanding fundamental mathematical ideas is more impactful than procedural fluency in these contexts. The limited influence of PK supports educational theories that emphasize conceptual understanding over rote learning in authentic learning scenarios.

The integration of EEG-based metrics into classroom practice opens new possibilities for precision education. For example, sustained elevations in theta activity—often associated with increased cognitive effort or attentional lapses—may indicate a need for targeted interventions, such as cognitive training focused on working memory, attentional control, and self-regulation. Recent systems like AttentivU provide real-time haptic biofeedback based on engagement levels, demonstrating improved learner attention and performance. Similarly, EEG-based adaptive systems have been developed for learners with special needs, such as children with ASD, where real-time attention tracking dynamically adjusts visual feedback to sustain focus. At a broader level, platforms like the Adaptive Neuro-Learning System (ANLS) combine EEG monitoring with intelligent tutoring systems, offering personalized instructional adjustments during online lectures. These examples illustrate how EEG-derived indicators—such as attention, engagement, or fatigue—can be operationalized to trigger timely instructional responses, supporting both general and individualized learning needs.

In the specific domain of mathematics education, EEG-informed teaching strategies could further enhance the learning of fractions—a domain known for persistent misconceptions and high cognitive demands. By incorporating rich, multimodal visual stimuli and differentiated interactive activities (e.g., the Diamond Paper or digital environments like Fraction Lab), educators can create adaptive learning scenarios that respond more effectively to students’ real-time cognitive states. Such environments not only support conceptual understanding but also help sustain engagement and reduce mental fatigue.

Because task order was fixed in individual sessions (Diamond Paper followed Fraction Lab), potential time-on-task and fatigue effects cannot be fully ruled out. In future studies, we will consider (i) two shorter appointments with randomized order across participants where feasible, or (ii) subgroup-level counterbalancing while preserving curricular prerequisites. We will also collect brief fatigue indices (e.g., a short self-report) and time-on-task proxies to help dissociate fatigue from task-specific difficulty.

## 5. Conclusions

Combining EEG data with assessments of conceptual and procedural fraction knowledge revealed clear distinctions in how students process mathematical tasks. Differences in spectral power across frequency bands highlighted varying levels of cognitive effort, attentional control, and task engagement.

Lower-performing students demonstrated increased activity in lower-frequency EEG bands, particularly during the final task of the session. This pattern may reflect heightened cognitive effort and signs of mental fatigue after extended engagement with demanding material. In contrast, students who performed at a higher level tended to show more stable activation across tasks, with greater involvement of higher-frequency bands typically linked to sustained attention and efficient cognitive processing. The use of EEG in educational research offers a valuable lens through which to examine how students engage with complex mathematical content. By observing real-time brain activity during task performance, educators and researchers can gain deeper insight into the cognitive demands placed on learners. This approach holds promise for informing more responsive instructional strategies that account for differences in attention, workload, and mental endurance across learners.

Building on the promising results of this study, the integration of portable EEG technology into broader educational contexts holds significant potential, particularly within scalable and IoT-enabled frameworks. The lightweight, wireless nature of devices like Muse 2 facilitates deployment across multiple school environments without the need for extensive infrastructure. With centralized data management systems, EEG recordings from multiple classrooms could be aggregated in real time, enabling large-scale analysis of cognitive engagement across diverse student populations. This scalability opens avenues for comparative studies between schools, regions, or educational approaches, providing policymakers and educators with actionable insights into how students respond to different instructional designs under authentic learning conditions.

Incorporating such EEG-based monitoring into edTech Internet of Things (IoT) platforms could further enhance adaptive learning environments. Real-time data streaming from wearable devices to cloud-based systems would allow for continuous assessment of student attention, mental workload, and cognitive fatigue. These physiological signals could then inform personalized feedback mechanisms or dynamically adjust digital learning content based on individual or group-level cognitive states. As part of a broader IoT ecosystem, this approach could integrate seamlessly with other sensors (e.g., eye tracking, motion detection) to enrich the understanding of student behavior and cognition. Ultimately, embedding neurophysiological data streams within educational technology platforms represents a transformative step toward evidence-based, learner-centered instruction at scale. One limitation of this study concerns the relatively small sample size, which may constrain the generalizability of the findings. Nonetheless, this constraint should be considered in the context of the study’s innovative focus. To the authors’ knowledge, this is the first research effort to integrate EEG monitoring with validated conceptual and procedural fraction assessments in a cohort of underage students within authentic educational environments.

Recruiting a larger number of participants in this age group presents practical and ethical challenges, including the need for parental consent and the constraints of school-based research. Despite this, the sample size employed is consistent with norms in the EEG literature. As reported by Morales et al. [72], in a review of 150 EEG studies published between 2011 and 2017, the average sample size per article was 29 (median = 22), and the average per group was 21 (median = 18), indicating that the present study’s sample meets widely accepted thresholds for statistical reliability. This work offers important contributions to the emerging field of educational neuroscience by providing novel evidence on how young learners engage with fractions at both cognitive and neural levels. While the current sample allows for meaningful insights and statistically supported conclusions, expanding the participant pool remains a priority for future research. Increasing the sample size in subsequent studies will not only enhance statistical power but also allow for deeper analysis of individual variability across diverse learner profiles, thereby strengthening the development of personalized educational interventions.

Taken together, the results underscore the promise of combining neurophysiological monitoring with multi-dimensional cognitive profiling to support individualized education. By capturing students’ brain activity during carefully selected tasks—ranging from diagnostic profiling (RODI, CU, PK) to fraction-specific learning activities (Fraction Lab, Diamond)—this study demonstrates how diverse data streams can converge to offer a rich understanding of each student’s learning profile. These insights open the door to designing adaptive learning pathways tailored to students’ cognitive characteristics and knowledge states. Future work will focus on translating these findings into practical educational tools and systems that can dynamically adjust instruction, feedback, and task design in real time, helping educators move toward a more personalized and inclusive vision of mathematics education.

## Figures and Tables

**Table 1:** ANOVA analysis of power between conditions.

Sensor	Frequency Band	*F*_4,134_	*p*-Value	Conditions
AF7	δ	3.17	0.016	Rodi-CK, Rodi-Diamond
AF7	θ	2.69	0.034	Rodi-Diamond
AF7	α	2.69	0.034	Rodi-CK, Rodi-Diamond

## Data Availability

The data presented in this study are available on request from the corresponding author.
